# Metabolic profiles of cysteine, methionine, glutamate, glutamine, arginine, aspartate, asparagine, alanine and glutathione in *Streptococcus thermophilus* during pH-controlled batch fermentations

**DOI:** 10.1038/s41598-018-30272-5

**Published:** 2018-08-20

**Authors:** Yali Qiao, Gefei Liu, Cong Leng, Yanjiao Zhang, Xuepeng Lv, Hongyu Chen, Jiahui Sun, Zhen Feng

**Affiliations:** Key Laboratory of Dairy Science, Ministry of Education, College of Food Science, Northeast Agricultural University, No.600, Changjiang Road, Harbin, 150030 China

## Abstract

Elucidating the amino acid (AA) metabolism patterns of *Streptococcus thermophilus* has important effects on the precise design of nitrogen sources for high-cell-density culture. Transcriptomics and metabolomics were combined to reveal the cysteine, methionine, glutamate, glutamine, arginine, aspartate, asparagine and alanine metabolic pathways in *S. thermophilus* MN-ZLW-002, including glutathione. The changes in the synthesis, consumption and concentration of AAs and their metabolites, as well as regulatory genes with time were revealed. The metabolism of L-cysteine, L-glutamate, L-aspartate and L-alanine generated some potential functional metabolites. The metabolism of methionine and glutamate generated potential harmful metabolites. *S. thermophilus* MN-ZLW-002 can synthesize glutathione. Some potential functional metabolites have similar biological functions, indicating that *S. thermophilus* can resist environmental stresses through multiple mechanisms. The expression of some key genes in synthesis pathway of AA indicated that cysteine, methionine, asparagine, aspartate, arginine and lysine were insufficient or imbalance between nutrient components. The accumulation of large amounts of AA metabolites might be the primary cause of the overconsumption of AAs and influence the growth of *S. thermophilus*. The present study revealed the metabolic profiles of abovementioned AAs as well as those of regulatory genes and metabolites. These results were beneficial to the precise design of nitrogen sources and regulation of functional metabolites for the high-cell-density culture of *S. thermophilus*.

## Introduction

*Streptococcus thermophilus* is widely used as a starter in the manufacturing of fermented dairy products. Based on changes in the preparation of the inoculums, starter cultures in increasingly concentrated forms have directly inoculated into the food matrix. In particular, the direct-vat-set starter has been widely used for the manufacturing of fermented dairy products^1^. For preparation of the direct-vat-set starter, high-cell-density culture is one of the foremost processes that strongly influence the quality of starter cultures. Starters are currently produced in cultures in which the pH is controlled at an optimal value through the continuous addition of sodium hydroxide^2^. *S*. *thermophilus* generates a large amount of lactic acid, which leads to a decrease in the intracellular pH because the intracellular H^+^ cannot be excreted on a timely basis. Furthermore, the accumulation of sodium lactate results in increases in the extracellular osmotic pressure. Thus, *S. thermophilus* encounters two stress factors simultaneously, namely, low intracellular pH and high extracellular osmotic pressure, particularly during the exponential growth phase^3,4^.

Lactic acid bacteria (LAB) have multiple amino acid (AA) requirements to synthesize proteins, provide precursors for the biosynthesis of AAs, nucleotides and vitamins, generate metabolic energy, control the intracellular pH and resist stresses^5,6^. Previous studies showed that glutamine, glutamate, arginine, aspartate and alanine are involved in protecting LAB against the damage caused by a low-pH environment^7–9^. Cysteine can restore oxygen tolerance in *Lactobacillus sanfranciscensis*^10^. Methionine plays a central role in the interconversion of sulfur-containing AAs^7^. Glutathione can protect LAB against oxidative stress, osmotic stress and acid stress^11^. However, the reasons for the functions of the abovementioned AAs and their metabolic pathways in *S. thermophilus* have not been fully studied. Our previous study showed that the consumption of some AAs notably exceeds the necessary amounts for the growth of *S. thermophilus*, implying a significant overconsumption of AAs^3,12^. Based on our results and those of previous studies, we proposed the following series of questions: (i) How are these AAs metabolized in *S. thermophilus*? (ii) What are the key genes involved in the regulation of the metabolism of these AAs? (iii) Which metabolites are generated? (IV) What pathway is responsible for the overconsumption of AAs? However, the metabolic pathways of various AAs in *S. thermophilus* during pH-controlled batch fermentations with a simulated high-density-culture environment have not systematically studied. The lack of reliable information on the metabolic patterns of AAs hinders the rational design of cultivation media and the optimization of relevant bioprocesses. Furthermore, the AA metabolism in *S. thermophilus* should be elucidated to better understand their use in pH-controlled batch fermentations.

To answer the abovementioned questions, transcriptomics and metabolomics were combined to investigate the metabolic profiles of these AAs in *S. thermophilus*. Clarification of these questions will aid the understanding of the mechanistic action of AAs on the growth of *S*. *thermophilus* and the design of cultivation media.

## Results

### Cysteine and methionine metabolism

**Figure 1:**
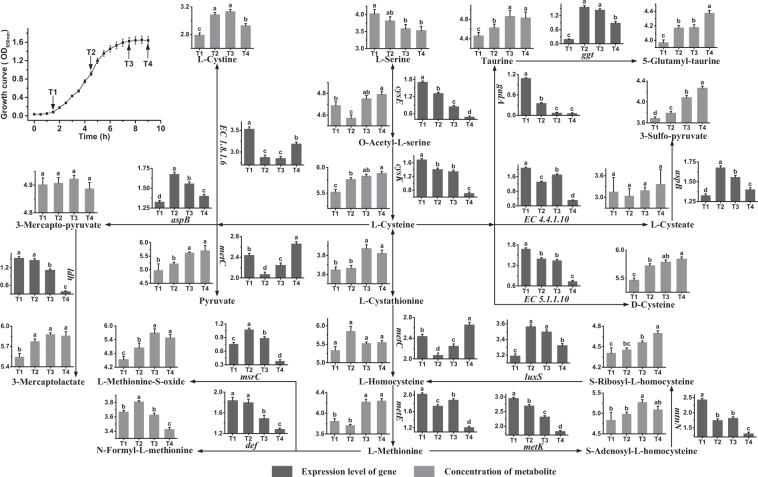
Changes in the expression levels of genes and concentrations of metabolites associated with the cysteine and methionine metabolic pathways in *S. thermophilus* MN-ZLW-002 (ST-MZ-2) during culture. The X axis displays the sampling time, and the Y axis displays Log10 FPKM and Log10 peak area for genes and metabolites, respectively. Values are mean ± SEM, *n* = 5. The bars with different superscripts are significantly different (*p* < 0.05).

Figure 1 shows the metabolic profiles of L-cysteine and L-methionine with their regulatory genes and metabolites. Table S1 lists the full names of all genes mentioned below. In the metabolic pathways of L-cysteine, genes *cysE* and *cysK* were down-regulated from T1 to T4. The results showed that the synthesis of L-cysteine from L-serine occurred mainly at T1. The expression levels of *gadA*, *EC 4.4.4.10*, *EC 5.1.1.10* and *EC 1.8.1.6* were high at T1, indicating that L-cysteine participated in the syntheses of some functional metabolites, such as L-cystine, taurine, L-cysteate and D-cysteine, mainly at T1. The expression levels of *aspB* and *metC* were high at T2 and T4. The results showed that the syntheses of 3-mercapto-pyruvate and pyruvate from L-cysteine occurred mainly at T2 and T4, respectively. These results also indicated that consumption of L-cysteine occurred mainly at T1, T2 and T4. Furthermore, the concentration of L-cysteine increased significantly from T1 to T4. The concentrations of some functional metabolites of L-cysteine, such as taurine, D-cysteine and pyruvate, increased significantly from T1 to T4, showing that their synthesis rates were higher than their consumption rates during this period. Gene *ggt* was expressed highly at T2 and T3, indicating that 5-glutamyl-taurine was synthesized mainly in these phases. And the concentration of this potential functional metabolite increased over time. The expression level of *aspB* also showed that the synthesis of 3-sulfo-pyruvate occurred mainly at T2. The concentration of 3-sulfo-pyruvate increased from T1 to T4. The concentrations of 5-glutamyl-taurine and 3-sulfo-pyruvate showed that their synthesis rates were higher than their consumption rates from T1 to T4.

In the metabolic pathways of L-methionine, gene *metE* was expressed highly at T1, indicating that L-methionine was synthesized mainly at T1. The concentration of L-methionine increased significantly from T2 to T3, showing that the rate of its synthesis plus uptake was higher than its consumption rate from T2 to T3. Furthermore, the high level of L-methionine at T3 might be caused by the up-regulation of *metE* from T2 to T3. Gene *msrC* was expressed highly at T2, showing that L-methionine converted to L-methionine-S-oxide mainly in this phase. The expression level of *def* was high at T1 and T2. The results indicated that the conversion of L-methionine to N-formyl-Lmethionine occurred mainly in these phases. L-methionine, S-adenosyl-L-homocysteine, S-ribosyl-L-homocysteine and L-homocysteine form a metabolic cycle. Gene *metK* was expressed highly at T1, indicating that L-methionine participated in the methionine cycle mainly in this phase. Furthermore, these results indicated that consumption of L-methionine occurred mainly from T1 to T2. The concentration of the functional metabolites of methionine, L-methionine-S-oxide, increased from T1 to T3. The results indicated that the synthesis rate of L-methionine-S-oxide was higher than its consumption rate during this period. A decrease in N-formyl-L-methionine was observed after T2. This decrease could be explained by reduced mRNA levels of *def* gene. The concentrations of S-adenosyl-L-homocysteine and S-ribosyl-L-homocysteine, the intermediates of the methionine cycle, were high at T3 and T4. Additionally, the expression levels of *metC* and *luxS* were complementary, thereby maintaining the concentration of L-homocysteine for the synthesis of L-methionine.

To conclude, most genes related to syntheses of cysteine, methionine and their metabolites were expressed highly in the early fermentation stage, especially at T1. The concentrations of cysteine, methionine and most of their functional metabolites were high in the late fermentation stage. The results showed that the rates of their synthesis plus uptake were higher than their consumption rates from T1 to T4.

### Glutamate, glutamine and arginine metabolism

**Figure 2:**
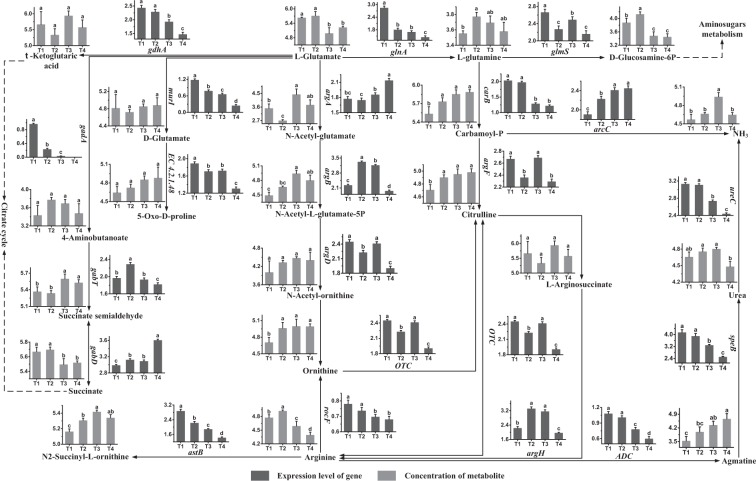
Changes in the expression levels of genes and concentrations of metabolites associated with the glutamate, glutamine and arginine metabolic pathways in ST-MZ-2 during culture. The X axis displays the sampling time, and the Y axis displays Log10 FPKM and Log10 peak area for genes and metabolites, respectively. Values are mean ± SEM, *n* = 5. The bars with different superscripts are significantly different (*p* < 0.05).

The metabolic profiles of glutamate, glutamine and arginine, as well as its regulatory genes and metabolites, are shown in Fig. 2. With respect to metabolism of glutamate, the expression levels of *gadA*, *murI* and *glnA* were high at T1, indicating that L-glutamate participated in the syntheses of 4-aminobutanoate, D-glutamate and L-glutamine mainly in this phase. Gene *gdhA* was expressed highly at T1 and T2, indicating that the formation of α-ketoglutaric acid from L-glutamate occurred mainly in these phases. Gene *argA* was expressed highly at T4, showing that the synthesis of N-acetyl-glutamate occurred mainly in this phase. These results indicated that consumption of glutamate occurred mainly at T1, T2 and T4. Additionally, the concentration of L-glutamate decreased significantly from T2 to T3. The results showed that the consumption rate of glutamate was higher than its uptake rate from T2 to T3. The expression levels of *gabT* and *gabD* were high at T2 and T4, respectively. The results indicated that the syntheses of succinate semialdehyde and succinate occurred mainly at T2 and T4, respectively. The concentration of potential toxic metabolite, succinate semialdehyde, was high at T3 and T4 and the concentration of succinate was high at T1 and T2. The up-regulation of *argA* from T2 to T3 might contribute to the accumulation of N-acetyl-glutamate during this period. N-acetyl-ornithine can produce ornithine, which was involved in arginine metabolism. Gene *argD* was expressed highly at T1 and T3, indicating that the synthesis of N-acetyl-ornithine occurred mainly in these phases. Additionally, the concentrations of 4-aminobutanoate, D-glutamate, 5-oxo-D-proline, α-ketoglutaric acid and N-acetyl-ornithine had no significant changes.

Gene *glmS* was expressed highly at T1 and gene *carB* was expressed highly at T1 and T2. These results indicated that L-glutamine participated in the syntheses of D-glucosamine-6P and carbamoyl-P mainly at T1 and T2. And consumption of L-glutamine occurred mainly in these phases. The concentration of L-glutamine increased significantly from T1 to T2, showing that the rate of its synthesis plus uptake was higher than its consumption rate during this period. The concentration of D-glucosamine-6P decreased significantly from T2 to T3, indicating that its consumption rate was higher than its synthesis rate during this period. The concentrations of carbamoyl-P and citrulline increased significantly from T1 to T2, showing that their synthesis rates were higher than their consumption rates during this period. Gene *argF* was expressed highly at T1 and T3, showing that the conversion of carbamoyl-P to citrulline occurred mainly in these phases.

The expression level of *argH* was high at T2 and T3, indicating that the synthesis of arginine from L-arginosuccinate occurred mainly in these phases. The concentration of arginine decreased after T2, showing that its consumption rate was higher than the rate of its synthesis plus uptake from T2 to T4. Gene *OTC* was expressed highly at T1 and T3. The results indicated that the synthesis of citrulline from ornithine, including the interconversion between citrulline and arginine, occurred mainly at T1 and T3. The expression levels of *astB* and *ADC* were high at T1 and T2, showing that the syntheses of N2-succinyl-L-ornithine and agmatine occurred mainly in these phases. These results indicated that consumption of arginine occurred mainly at T1 and T2. The concentrations of N2-succinyl-L-ornithine and agmatine increased significantly from T1 to T4. A decrease in urea was observed after T3. This decreased could be caused by down-regulation of *ureC* from T3 to T4. With respect to the synthesis of NH_3_, the expression levels of *arcC* and *ureC* were complementary, thereby maintaining the concentration of NH_3_. The concentration of NH_3_ decreased from T3 to T4. The results indicated that the consumption rate of NH_3_ was higher than its uptake rate after T3.

The present results indicated that the syntheses of L-glutamate and arginine mainly occurred in the late-lag phase and exponential phase, respectively. The expression levels of most genes in the glutamate, glutamine and arginine metabolic pathways were high in the early fermentation stage. The changes in the concentrations of L-glutamate and arginine showed that their consumption rates were higher than their synthesis rates in the late fermentation stage.

### Aspartate and asparagine metabolism

**Figure 3:**
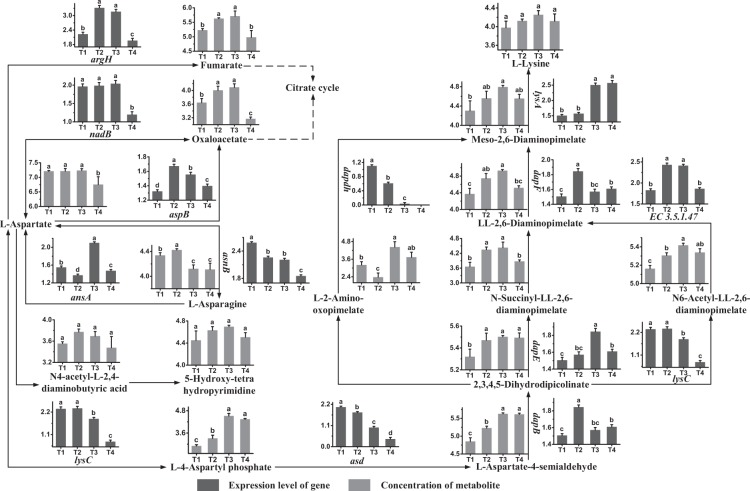
Changes in the expression levels of genes and concentrations of metabolites associated with the aspartate metabolic pathway in ST-MZ-2 during culture. The X axis displays the sampling time, and the Y axis displays Log10 FPKM and Log10 peak area for genes and metabolites, respectively. Values are mean ± SEM, *n* = 5. The bars with different superscripts are significantly different (*p* < 0.05).

The metabolic profiles of aspartate and asparagine, with its regulatory genes and metabolites, are shown in Fig. 3. The expression levels of *asnA* and *asnB* were high at T3 and at T1, respectively. These results showed that aspartate was mainly synthesized at T3 and that interconversion between aspartate and asparagine occurred mainly at T1. The expression levels of *argH* and *aspB* were high at T2 and T3 and at T2, respectively. These results indicated that aspartate was mainly consumed at T2 and T3 and that the formation of furmarate and oxaloacetate, the intermediates in the citrate cycle, from L-aspartate occurred mainly in these phases. The concentrations of aspartate and asparagine decreased significantly from T3 to T4 and from T2 to T3, respectively. The concentrations of fumarate and oxaloacetate decreased significantly from T3 to T4, indicating that their synthesis rates were lower than their consumption rates during this period. The concentrations of N4-Acetyl-2,4-diaminobutyric acid and 5-Hydroxy-tetrahydropyrimidine did not changed significantly during growth.

The present study showed that aspartate generated lysine through three pathways. 2,3,4,5-Dihydrodipicolinate, LL-2,6-diaminopimelate and Meso-2,6-diaminopimelate were key intermediates. The expression levels of *dapB* and *EC 3.5.1.47* were high at T2 and at T2 and T3, respectively. The results indicated that the syntheses of 2,3,4,5-dihydrodipicolinate and LL-2,6-diaminopimelate occurred mainly in these phases. The concentration of 2,3,4,5-dihydrodipicolinate increased from T1 to T2. The up-regulation of *EC 3.5.1.47* from T1 to T3 might contribute to the accumulation of LL-2,6-diaminopimelate during this period. The expression levels of *dapdh* and *dapF* were high at T1 and T2, respectively. The results showed that the syntheses of Meso-2,6-diaminopimelate from L-2-amino-oxopimelate and LL-2,6-diaminopimelate occurred mainly in these phases. A significant increase in the concentration of Meso-2,6-diaminopimelate was observed from T1 to T3. This increased could be explained by the up-regulation of *dapF* during this period. Gene *lysA* was expressed highly at T3 and T4, indicating that the synthesis of L-lysine occurred mainly in these phases. Additionally, there was no significant change in the concentration of lysine from T1 to T4.

The present results revealed that syntheses of aspartate and lysine occurred mainly at T3 and T4. Most genes related to the metabolism of aspartate and asparagine were down-regulated from T2 to T4. The consumption rates of aspartate, asparagine and most metabolites were higher than their synthesis rates during the late fermentation stage. The synthesis rates of most metabolites of lysine were higher than their consumption rates during the exponential phase.

### Alanine and glutathione metabolism

**Figure 4:**
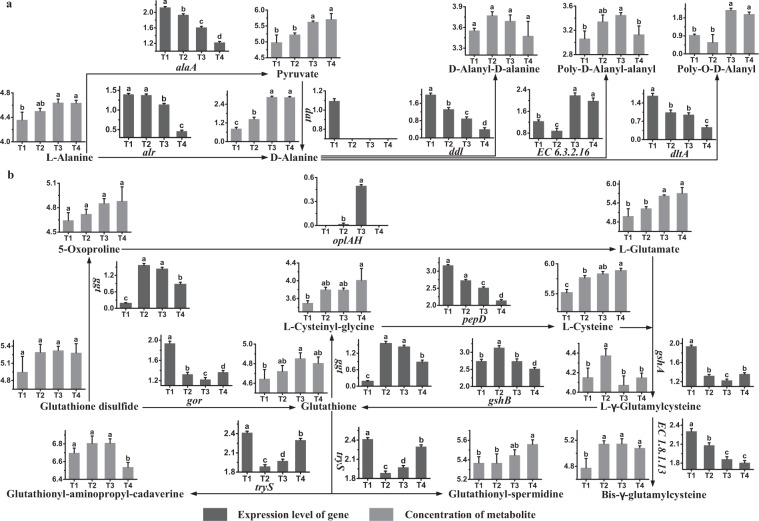
Changes in the expression levels of genes and concentrations of metabolites associated with the alanine (**a**) and glutathione (**b**) metabolic pathway in ST-MZ-2 during culture. The X axis displays the sampling time, and the Y axis displays Log10 FPKM and Log10 peak area for genes and metabolites, respectively. Values are mean ± SEM, *n* = 5. The bars with different superscripts are significantly different (*p* < 0.05).

The metabolic profiles of L-alanine and glutathione, as well as their regulatory genes and metabolites, are shown in Fig. 4a,b, respectively. The expression levels of *alaA* and *alr* were high at T1 and at T1 and T2, respectively. The results indicated that pyruvate and D-alanine were mainly synthesized in these phases, and L-alanine was mainly consumed at the same time. The concentrations of L-alanine and D-alanine were high at T3 and at T4, respectively. The expression of *dat* was undetectable after T1, indicating that pyruvate was involved in D-alanine synthesis mainly at T1, and synthesis of D-alanine from L-alanine did not meet the requirements at T1. The expression levels of *dltA*, *ddl* and *EC 6.3.2.16* were high at T1 and at T3 and T4. The results indicated that poly-O-D-alanyl, D-alanyl-D-alanine and poly-D-alanyl-alanyl were synthesized mainly in these phases. Meanwhile, D-alanine was mainly consumed at the same time. A significant decrease in the concentration of Poly-D-Alanyl-alanyl was observed after T3, indicating that its consumption rate was higher than its synthesis rate from T3 to T4.

The expression levels of *gor* and *gshB* were high at T1 and T2, respectively. The results indicated that synthesis of glutathione occurred mainly in these phases. Gene *ggt* was expressed highly at T2 and T3, showing that the formation of L-cysteinyl-glycine from glutathione occurred mainly in these phases. Gene *tryS* was expressed highly at T1, showing that the synthese of glutathionyl-spermine and glutathionyl-aminopropyl-cadaverine from glutathione occurred mainly in this phase. These results revealed that glutathione was mainly consumed from T1 to T3. The concentration of glutathione increased from T1 to T3. The concentration of glutathionyl-spermine was high at T4. Genes *gshA* and *EC 1.8.1.13* were expressed highly at T1, indicating that L-γ-glutamylcysteine and bis-γ-glutamylcysteine were synthesized mainly at T1. There was no significant change in the concentration of bis-γ-glutamylcysteine from T2 to T4. The concentration of L-γ-glutamylcysteine decreased significantly from T2 to T4, indicating that its consumption rate was higher than its synthesis rate after T2. The expression level of *oplAH* was high at T3, indicating that the synthesis of L-glutamate from 5-oxoproline occurred mainly in this phase. And the high expression of *pepD* at T1 and T2 indicated that the synthesis of L-cysteine from L-cysteinyl-glycine occurred mainly in these phases.

The present results showed that D-alanine and glutathione were synthesized mainly in the early fermentation stage. Genes in the alanine and glutathione metabolic pathways were expressed highly in the early fermentation stage. The synthesis rates of alanine, glutathione and most of their metabolites were higher than their consumption rates in the exponential phase.

## Discussion

Shortages of cysteine and methionine would completely prevent the growth of *S. thermophilus*^13^. The expression of the *cysE*, *cysK*, *metC*, *metE* and *metK* observed in this study indicated that serine was involved in the synthesis of cysteine and cysteine was involved in the synthesis of methionine. The expression levels of *cysE*, *cysK* and *metE* indicated that intracellular concentrations of cysteine and methionine were insufficient at T1. Meanwhile, there were cysteine and methionine in the media. The results of the present study indicate that rate of their uptake did not meet their requirement. In terms of high-cell-density cultivation, distinct ratios of AAs in the growth media were very important for biomass optimization^14^. The imbalance between nutrient components may lead to the above phenomenon. Additionally, the expression levels of *metC* and *luxS* were complementary, thereby maintaining the concentration of homocysteine for the synthesis of methionine. D-cysteine, as a component of cell wall peptidoglycans, is indispensable for bacterial growth. The present study showed that *S. thermophilus* MN-ZLW-002 (ST-MZ-2) has the ability to synthesize taurine. This metabolite regulates an unusual number of biological phenomena, including cell growth and viability, cytoprotective activity, membrane structure and protein phosphorylation, but its most important function is its osmoregulatory activity, which reflect its role as a key organic osmolyte^15^. The concentration of taurine was increased with extracellular osmotic pressure. Taurine might enhance the capacity of ST-MZ-2 to resist osmotic pressure. Additionally, we inferred that 5-glutamyl-taurine has functions similar to those of taurine. L-cysteate can function as a sulfur and nitrogen source for the growth of bacteria^16^. Protein synthesis is initiated by formylmethionine in prokaryotes^17^. In this study, the changes in concentration of N-formyl-L-methionine indicated that protein synthesis decreased gradually. L-methionine-S-oxide impairs molecular integrity and functionality^18^. In this study, the changes in its concentration indicated that impairments of molecular integrity and functionality were enhanced gradually. These could be an inhibiting factor in the growth of ST-MZ-2.

L-glutamate metabolites and D-glutamate are indispensable for bacterial growth as components of cell wall peptidoglycans. A previous study indicated that succinate semialdehyde might be a toxic intermediate in *Escherichia coli*^19^. The changes in the concentration of succinate semialdehyde showed that this could be an inhibiting factor in the growth of ST-MZ-2. Glutamate, glutamine and arginine improve the acid resistance of LAB^9^. In this study, glutamine and arginine improved the acid resistance of ST-MZ-2 by generating NH_3_. The expression levels of *ureC* and *arcC* were complementary, indicating that glutamine and arginine improved the acid resistance of ST-MZ-2 at different stages of growth. The results of the present study indicated that glutamate and glutamine were involved in the synthesis of arginine. In a bacterium auxotrophic for arginine, arginine synthesis occurs through a de novo pathway that uses N-succinyl-L-ornithine. The results of the current study showed that ST-MZ-2 does not have the ability to synthesize arginine through a de novo pathway. The expression levels of *astB* and *ADC*, and changes in arginine concentration is consistent with our previous research results^3,12^. N-acetyl-ornithine has been found to protect against osmotic stress in some microorganisms^20^, but whether N-acetyl-ornithine helps ST-MZ-2 resist osmotic stress needs further study. Additionally, D-glucosamine-6P is involved in amino sugar metabolism and the changes of concentration indicated that D-glucosamine-6P was insufficient at the late-exponential growth phase.

Fumarate and oxaloacetate are involved in the citrate cycle. The expression levels of their synthesis genes and changes of their concentrations indicated that aspartate was not found at sufficient amounts for their synthesis at the stationary phase. A previous study showed that aspartate was hardly consumed and should thus be produced from asparagine^12^. The overconsumption of asparagine might be necessary to maintain an effective transfer of amino groups. Energetically, the transport of asparagine was notably more efficient than that of aspartate; moreover, asparagine is likely preferentially directed toward the production of aspartate^21^. Thus, we inferred that the major role of asparagine was the synthesis of aspartate and that the role of *asnB* was to maintain a suitable concentration of aspartate. N4-Acetyl-2,4-diaminobutyric acid and 5-Hydroxy-tetrahydropyrimidine played a very important role in regulating the osmotic pressure and stabilizing the protein structure of cells^22^. They might enhance the capacity of ST-MZ-2 to resist osmotic pressure. Most bacteria can synthesize diaminopimelate and lysine from aspartate, which was a major source of lysine in bacteria and was essential for cell growth and proliferation^23,24^. Diaminopimelate and lysine are essential components of the peptidoglycan cell wall of most gram-positive bacteria^23^. The present result showed that aspartate was involved in the synthesis of lysine, indicating that lysine was found at insufficient level, especially from end-exponential growth phase to stationary phase.

Alanine metabolism plays an important role in the synthesis and defense of the cell wall^25^. The *dltA* gene can be involved in the alanylation of phosphate residues in gram-positive bacteria. D-alanylated phosphate regulates autolysin transport, cationic homeostasis, nutrients, proteins and the transport of enveloped proteins. Poly-O-D-alanyl is the major component of teichoic acid, and D-alanyl-D-alanine is used for the synthesis of peptidoglycan^26^. Poly-D-alanyl-alanyl is also involved in the synthesis of phosphoric acid and peptidoglycans in *E. coli* and *Bacillus subtilis*. In this study, D-alanine generated abovementioned functional metabolites. Previous results revealed that the bacteria preferred to use D-alanine^27^. Thus, D-alanine should be added to media instead of L-alanine during the culture of ST-MZ-2. ST-MZ-2 can synthesize glutathione, as was demonstrated in this study, and a similar result was obtained in *S. thermophilus* ATCC 19258^28^. Glutathione plays diverse roles in biological systems; specifically, it has antioxidative and detoxifying activities and enhances the resistance of LAB against various stresses, including oxidative, acid and osmotic stresses^29^. The present study showed that glutathione consumption occurs mainly at the stationary phase, which is the phase in which ST-MZ-2 encounters high environmental stress. Thus, glutathione played important roles in helping ST-MZ-2 resist environmental stresses. L-γ-glutamylcysteine could enhance the glutathione content and increase the scavenging of free radicals to prevent oxidative damage^30^. Bis-γ-glutamylcysteine might play a crucial role in the detoxification of highly reactive oxygen and nitrogen species^31^. Glutathionyl-spermidine more effectively prevents DNA damage induced by free radicals or oxidants^32^. Glutathionyl-spermidine is also involved in cell proliferation and differentiation in *E. coli*^33^. The genes and metabolites involved in the physiological roles of glutathione are unclear. Based on the results of the current study, we inferred that the physiological roles of glutathione could be attributed mainly to its metabolites. Additionally, LAB have the capacity to synthesize glutathione, which is an important property of cells that allow them to withstand harsh processing treatments^29^.

Previous studies have shown that the consumption of AAs exceeded the amounts necessary for the growth of *L. lactis* and *S. thermophilus*, implying a significant overconsumption of AAs^3,12,21^. The findings obtained in the present study indicated that the overconsumption of AAs might be mainly attributed to the production of large amounts of end-products and intermediates of the AA metabolism. The functions of many of the metabolites investigated in this study, particularly in *S. thermophilus*, were unknown. Thus, more in-depth studies are needed to provide further data to clarify their functions. The harmful metabolites could not be removed by the bacteria, indirectly blocking cell proliferation^34^. As shown in the present study, the concentration of most of metabolites was high at T3 and T4, the accumulation of some potentially harmful metabolites might be an important factor for the termination of LAB growth. According to the present results, the levels of cysteine, methionine, asparagine, aspartate, arginine and lysine were insufficient or imbalance between nutrient components. Meanwhile, consumption time of these AAs was also revealed. The ideal media for high-cell-density cultivation should supply various nutrients with more suitable ratios for the growth of LAB, since the synthesis of nutrients requires the consumption of other nutrients, with energy consumption and production of useless intermediate metabolites^35^. Therefore, supplementation of these AAs by feeding strategies or increasing their original concentration might aid the growth of ST-MZ-2. Additionally, some potential functional metabolites have similar biological functions, indicating that LAB can resist environmental stresses through multiple mechanisms.

In conclusion, the present study provides a comprehensive overview of the changes in the transcriptomes and metabolomes of cysteine, methionine, glutamate, glutamine, arginine, aspartate, asparagine, alanine and glutathione in *S. thermophilus* during pH-controlled batch fermentations. Furthermore, the genes involved in the regulation of the abovementioned AAs metabolic pathways and their metabolites were elucidated. The roles of many metabolites of abovementioned AAs and the design of a properly balanced culture medium based on the AA metabolism will be the focus of future research.

## Materials and Methods

### Strains, culture conditions and fermentation experiments

ST-MZ-2 was obtained as described in a previous study^36^. Culture stocks were prepared in 10% w/v sterile reconstituted skim milk containing 10% glycerol and then stored at −80 °C. Before use, eight subcultivations were performed in chemically defined medium (CDM) to obtain a stable growth response for 12 h at 42.5 °C. The CDM was prepared according to the method described by Lahtvee *et al*.^21^. Batch fermentations were performed in a 10-L Biotech-7000 bioreactor (Shanghai Baoxing, Shanghai, China) containing 7 L of CDM. The culture was centrifuged (10000 g, 10 min, 4 °C), and the cells were washed twice with PBS buffer (50 mmol/L, pH 6.5) and then inoculated into the bioreactor. The temperature and rotation speed were fixed to 42.5 °C and 200 rev/min, respectively. The pH was maintained at 6.25 through automatic addition of 1 mol/L NaOH. Samples were taken at four different time points representing late-lag (T1, OD_650nm_ = 0.09), mid-exponential (T2, OD_650nm_ = 0.9), late-exponential (T3, OD_650nm_ = 1.5) and stationary phases (T4, OD_650nm_ = 1.6) (Fig. 1). At the indicated time points, the cultures were centrifuged (12000 g, 4 °C, 15 min), the supernatant was discarded, and the pellet was snap-frozen in liquid nitrogen. Each culture condition was repeated five times.

### Transcriptomic analysis

RNA isolation and library construction for transcriptome analysis were performed as described by Zeng *et al*.^37^ with some modifications. In this study, the total RNA quantity and purity were assessed using Bioanalyzer 2100 and RNA 6000 Nano LabChip Kit (Agilent, Santa Clara, CA, USA) with an RNA integrity number of 7.0. Approximately 5 μg of total RNA was used for the depletion of ribosomal RNA according to the Ribo-Zero Gold rRNA Removal Kit manual (Illumina, San Diego, CA, USA). mRNA was incubated with fragmentation buffer in a pre-heated tube for 5 min at 94 °C and then fragmented into short fragments. cDNA was synthesized using the mRNA fragments as templates. Short fragments were purified and resolved with EB buffer for end reparation and single nucleotide A (adenine) addition. After that, the short fragments were connected with adapters. The suitable fragments were selected for the PCR amplification as templates. During the QC steps, Agilent 2100 Bioanaylzer and ABI StepOnePlus Real-Time PCR System were used in quantification and qualification of the sample library. The average insert size for the final single-end cDNA libraries was 250 bp (±50 bp). We then performed single-read sequencing (50 bp) using an Illumina HiSeq2500 instrument following the manufacturer’s recommended protocol. The transcriptome data were analyzed according to method described by Shen *et al*.^38^ with the exception that clean reads with a length of 36 nt were obtained. The following analysis was based on clean reads, which are generated by filtering raw reads. Transcriptome de novo assembly was carried out with Trinity, a short reads assembling program. Unigenes, the result sequences of Trinity, were annotated with the databases. The calculation of Unigene expression uses FPKM method (Fragments Per kb per Million reads). For DEG analysis, the False Discovery Rate (FDR) is used to correlate p-values. Based on the FPKM levels of different samples, we chose those with an FDR ≤ 0.001 and a ratio larger than 2 as differentially expressed genes.

### Metabolomic analysis

For the extraction of intracellular metabolites, the pellet (25 mg) was resuspended in 800 μL of methanol and water (1:1, v/v), and metabolites were extracted using a TissueLyser (60 Hz) for 5 min at room temperature. Cell debris was removed by centrifugation (25000 g, 4 °C, 20 min), and the samples (200 μL) were dried in a vacuum concentrator until the volume was less than 10 μL and then resuspended in ultrapure H_2_O to a final volume of 50 μL. One microliter of 0.5 mmol/L AZT was added as an internal standard. Liquid chromatography tandem mass spectrometry data were acquired on a 2777 C UPLC system (Waters, USA) coupled to a Xevo G2-XS Q-TOF mass spectrometer (Waters, USA). First, all chromatographic separations were performed using an ultra-performance liquid chromatography (UPLC) system (Waters, USA), and an ACQUITY UPLC BEH C18 column (100 mm * 2.1 mm, 1.7 μm, Waters, USA) was used for the reversed-phase separation. The column oven was maintained at 50 °C, and the flow rate was 0.4 mL/min. The mobile phases consisted of water (A) and acetonitrile (B), both with 0.1% formic acid. The elution program was the following: 0~2 min, 100% phase A; 2~11 min, 0% to 100% B; 11~13 min, 100% B; and 13~15 min, 100% A. The injection volume was 10 μL.

A high-resolution tandem mass spectrometer (SYNAPT G2 XS Q-TOF, Waters, USA) was used to detect the metabolites the eluted from the column. The Q-TOF was operated in both the positive and negative ion modes. For the positive ion mode, the capillary and sampling cone voltages were set to 0.25 kV and 40 V, respectively. For the negative ion mode, the capillary and sampling cone voltages were set to 2 kV and 40 V, respectively. The mass spectrometry data were acquired in the centroid MSE mode. The TOF mass range was from 50 to 1200 Da, and the scan time was 0.2 s. For the MS/MS detection, all precursors were fragmented with 20~40 eV, and the scan time was 0.2 s. During the acquisition, the LE signal was acquired every 3 s to calibrate the mass accuracy. Furthermore, to evaluate the stability of the LC-MS instrument during the acquisition, a quality control sample was acquired every 10 samples. The data were processed using Progenesis QI (version 2.2).

### Function elucidation and metabolic network construction

The KEGG (http://www.genome.jp/kegg/) and COG databases (http://www.ncbi.nlm.nih.gov/COG/) were used to classify and group the identified genes and metabolites.

### Data availability

The datasets generated during and/or analyzed during the current study are available from the corresponding author on reasonable request.

## Electronic supplementary material

Table S1
