# Supplementary material for: Metabolic profiles of cysteine, methionine, glutamate, glutamine, arginine, aspartate, asparagine, alanine and glutathione in Streptococcus thermophilus during pH-controlled batch fermentations
Source: Sci Rep. 2018 Aug 20;8:12441. doi: 10.1038/s41598-018-30272-5 (PMC6102215; doi:10.1038/s41598-018-30272-5)
Supplement: Supplementary file 1 — Table S1 [file 41598_2018_30272_MOESM1_ESM.docx]

**Metabolic profiles of cysteine, methionine, glutamate, glutamine, arginine, aspartate, asparagine, alanine and glutathione in *Streptococcus thermophilus* during pH-controlled batch fermentations**

Yali Qiao, Gefei Liu, Cong Leng, Yanjiao Zhang, Xuepeng Lv, Hongyu Chen, Jiahui Sun & Zhen Feng^*^

Table S1. List of the full name and the abbreviation of genes.

| Abbreviation | Gene name |
| --- | --- |
| *cysE* | O-acetyltransferase |
| *cysK* | Cystine synthase A |
| *gadA* | Glutamate decarboxylase |
| *EC 4.4.4.10* | Cysteine (sulfite) lyase |
| *EC 5.1.1.10* | Amino-acid racemase |
| *EC 1.8.1.6* | Cystine reductase |
| *aspB* | Aspartate aminotransferase |
| *metC* | Cystathionine beta-lyase |
| *ggt* | Gamma-glutamyltranspeptidase |
| *ldh* | L-lactate dehydrogenase |
| *metE* | 5-methyltetrahydropteroyltriglutamate-homocysteine methyltransferase |
| *metK* | S-adenosylmethionine synthetase |
| *msrC* | L-methionine (R)-S-oxide reductase |
| *def* | Formylmethionine deformylase |
| *mtnN* | Adenosylhomocysteine nucleosidase |
| *luxS* | S-ribosylhomocysteine lyase |
| *gdhA* | Glutamate dehydrogenase |
| *murI* | Glutamate racemase |
| *argA* | Amino-acid N-acetyltransferase |
| *glnA* | Glutamine synthetase |
| *gabT* | 4-aminobutyrate aminotransferase |
| *gabD* | Succinate-semialdehyde dehydrogenase |
| *EC4.2.1.48* | D-glutamate cyclase |
| *argA* | Amino-acid N-acetyltransferase |
| *argB* | Acetylglutamate kinase |
| *argD* | Acetylornithine aminotransferase |
| *carB* | Carbamoyl-phosphate synthase |
| *glmS* | Glucosamine-fructose-6-phosphate aminotransferase (isomerizing) |
| *arcC* | Carbamate kinase |
| *argF* | Ornithine carbamoyltransferase |
| *argH* | Argininosuccinate lyase |

Table S1. *cont*.

| Abbreviation | Gene name |
| --- | --- |
| *OTC* | Arginine deiminase |
| *astB* | Succinylarginine dihydrolase |
| *ADC* | Acetoacetate decarboxylase |
| *speB* | Agmatinase |
| *ureC* | Urease subunit alpha |
| *ansA* | Aspartate--ammonia ligase |
| *asnB* | Asparagine synthase |
| *nadB* | L-aspartate oxidase |
| *dapB* | 4-hydroxy-tetrahydrodipicolinate reductase |
| *EC 3.5.1.47* | N-acetyldiaminopimelate deacetylase |
| *dapdh* | Diaminopimelate dehydrogenase |
| *dapF* | Diaminopimelate epimerase |
| *lysA* | Diaminopimelate decarboxylase |
| *alaA* | Alanine-synthesizing transaminase |
| *alr* | Alanine racemase |
| *dat* | D-alanine transaminase |
| *dltA* | D-alanine--poly(phosphoribitol) ligase subunit 1 |
| *ddl* | D-alanine-D-alanine ligase |
| *EC 6.3.2.16* | D-alanine---alanyl-poly(glycerolphosphate) ligase |
| *gor* | Glutathione reductase (NADPH) |
| *gshB* | Glutathione synthase |
| *tryS* | Trypanothione synthetase/amidase |
| *oplAH* | 5-oxoprolinase (ATP-hydrolysing) |
| *pepD* | Dipeptidase D |
| *gshA* | Glutamate-cysteine ligase |
| *EC 1.8.1.13* | bis-gamma-glutamylcystine reductase |
